# Draft Genome Sequence of *Gulbenkiania mobilis* Strain MB1, a Sulfur-Metabolizing Thermophile Isolated from a Hot Spring in Central India

**DOI:** 10.1128/genomeA.01295-15

**Published:** 2015-11-19

**Authors:** Rituja Saxena, Nikhil Chaudhary, Darshan B. Dhakan, Vineet K. Sharma

**Affiliations:** Metagenomics and Systems Biology Laboratory, Department of Biological Sciences, Indian Institute of Science Education and Research, Bhopal, Madhya Pradesh, India

## Abstract

This paper reports the draft genome sequence of the proteobacterium *Gulbenkiania mobilis* strain MB1, a sulfur-metabolizing thermophile isolated from a hot spring located in Pachmarhi, India. This study reports the first draft genome sequence of any species from the genus *Gulbenkiania*.

## GENOME ANNOUNCEMENT

Thermophiles are of biotechnological interest due to their resistance to denaturing agents and for their production of thermostable enzymes (1). Few bacterial species of the genus *Gulbenkiania* have been isolated from different aquatic environments, such as hot springs and treated municipal wastewater (2, 3). *Gulbenkiania mobilis* strain MB1, reported in this study, was isolated from Chhoti Anhoni, a hot spring located near the hill station Pachmarhi in the state of Madhya Pradesh, India (22.65°N, 78.36°E). The on-site temperature, pH, and total dissolved solids concentration (TDS) of the hot spring were recorded to be 43.5°C, 7.8, and 590 parts per million (ppm), respectively.

*G. mobilis* strain MB1 was isolated on tryptone yeast extract dextrose agar with known composition (0.6% casein enzymatic hydrolysate, 0.3% yeast extract, 0.5% dextrose powder, and 2% agar [pH 7.5]), supplemented with 0.125% dipotassium hydrogen phosphate. The colonies were observed at a temperature range between 45 and 55°C within 24 to 48 h of incubation, under aerobic conditions. The colonies were small, round, opaque, and light brown in color with regular margins, and the bacterium was observed to be Gram negative and rod-shaped. Genomic DNA from pure culture was extracted using a phenol-chloroform extraction method.

The 16S rRNA sequence of MB1 showed 99% sequence identity with *G. mobilis* strain E4FC31, previously isolated from treated municipal wastewater, indicating MB1 to be a novel strain of *G. mobilis*. Genome sequencing of *G. mobilis* strain MB1 was performed with the Illumina NextSeq 500 platform (Illumina, San Diego, CA, USA) using a 150-bp length paired-end sequencing method. A total of 652 Mb of data (fastq format) were produced by sequencing, and 2,127,212 reads were used for assembly using SPAdes 3.5.0 (4). The draft genome size of *G. mobilis* strain MB1 is 3,312,197 bp in 187 contigs (length, ≥300 bp), with an *N*_50_ contig length of 106,255 bp and an average length of 17,712 bp. The G+C content of the draft genome is 62%. Gene prediction was performed using Glimmer version 3.02 (5). tRNAscan-SE version 1.3.1 and RNAmmer version 1.2 were used to identify tRNAs and rRNAs, respectively (6, 7). A total of 2,905 protein-coding genes, 39 tRNAs, and 1 rRNA operon with 16S, 23S, and 5S rRNAs were predicted. The coding sequences (CDSs) were searched against the NCBI NR using BLAST package (version 2.2.26; NCBI) for functional annotation and the KEGG GENES database using the KEGG Automated Annotation Server (KAAS) for identifying metabolic pathways (8–10). The MB1 genome contains genes for sulfur metabolism proteins, such as 3′-phosphoadenosine 5′-phosphosulfate, which is required for the assimilation of sulfate to sulfide. The *sox* gene for sulfur-oxidizing proteins and *soxD* encoding cytochrome *c* are also present, indicating that MB1 can utilize sulfur in addition to oxygen as the terminal electron acceptor in the electron transport chain. A more detailed genomic analysis of strain MB1 will be performed in the future to provide a deeper functional insight into this species.

### Nucleotide sequence accession numbers.

This whole-genome shotgun project has been deposited at DDBJ/EMBL/GenBank under the accession no. LIVN00000000. The version described in this paper is version LIVN01000000.
